# Investigation of the Application of *miR10b* and *miR135b* in the Identification of Semen Stains

**DOI:** 10.1371/journal.pone.0137067

**Published:** 2015-09-10

**Authors:** Dayue Tong, Yi Jin, Tianyu Xue, Xiaoyan Ma, Jinxiang Zhang, Xueling Ou, Jianding Cheng, Hongyu Sun

**Affiliations:** 1 Forensic Biology Section, Zhongshan Schoool of Medicine, Sun Yat-Sen University, Guangzhou, 510080, China; 2 Department of Pathology, the Third Affiliated Hospital, Sun Yat-Sen University, Guangzhou, 510630, China; Xi'an Jiaotong University School of Medicine, CHINA

## Abstract

To evaluate the identification method using the microRNA markers *miR10b* and *miR135b* to distinguish semen stains from menstrual blood, peripheral blood, vaginal fluid and so on body fluid stains. The expression levels of *miR10b* and *miR35b* in semen stains and menstrual blood and so on were detected utilizing a real-time quantitative PCR technique with a specific fluorescence-labeled TaqMan probe. *RNU6b* was used as the internal reference gene; the difference in their expression was analyzed, and the specificity, sensitivity, and detection capability of the techniques were evaluated. The expression of *miR10b* and *miR135b* in semen stains was significantly higher than that of other body fluid stains, with a mean value of ΔCт from-6 to-7. However, it ranged from-2 to-4 for other body fluid stains. The initial criteria for judging which semen stains can be identified were determined by analyzing the research results. When the threshold value was set to 0.04, the C_T_ value could be detected in the target genes *miR10b*, *miR135b* and in the internal reference gene *RNU6b*, and C_T_ values are<40, ΔC_T_[10b-U6]<-5.5, and ΔC_T_[135b-U6]<-6, respectively, and the semen stain could be identified. The expression levels of *miR10b* and *miR135b* are higher in semen with strong tissue specificity; thus, they can be used to differentiate semen stains from other body fluid stains in forensic science.

## Introduction

MicroRNAs (miRNAs) are a type of endogenous non-coding RNA that has a regulatory function in eukaryotes. The length of miRNAs molecule is approximately 20 to 25 nucleotides. They are generated by Dicer enzyme shearing single-stranded RNA precursors, which contain approximately 70 to 90 nucleotides with a hairpin structure [1, 2]. The miRNAs participate in a variety of regulatory pathways, including growth, virus defense, haematopoiesis, organogenesis, cell proliferation, apoptosis, and fat metabolism, and so on. Recently, they have also been shown to play an important role in tumorigenesis and other related fields. Therefore, the study and identification of miRNAs has become a hot topic in biology [3–5]. It has been found that the nucleotide sequence of a miRNA is short and that its structure is relatively stable, and a large number of studies have demonstrated that miRNAs do not easily undergo degradation. This particular feature has important significance and may aid in detection with problematic forensic evidence samples, particularly for degraded and old samples. As a new type of body fluid identification marker, miRNAs can be used to identify body fluids stains with difficult degradation characteristics [6–9]. It has been reported that the miRNA markers *miR10b* and *miR135b* are found in the seminal plasma or in exfoliated cells of the male reproductive tract, with an expression level higher than that of other body fluids [7]. This study was performed to evaluate the use of *miR10b* and *miR135b* to identify semen stains from among other bodily fluids stains. These miRNA markers may lead to a novel semen stain identification method.

## Materials and Methods

Semen specimens of forty adult males (with normal semen) and five adult male patients with azoospermia were collected. Samples were obtained from the first Affiliated Hospital, Sun Yat-Sen University. The semen samples were used to create semen stains according to forensic operational processes. The 100μl mix liquefied semen was taken, and smeared on the sterile gauze, natural airing, including preparation at 25°C under dry and dark conditions and drying under natural conditions. They were stored at 25°C in dry and dark conditions for one day, one month, two months, three months, six months, or twelve months. Furthermore, RNA was extracted from all semen stains with TRIzol Reagent (Invitrogen, Carlsbad, USA) according to manufacturer’s instructions. In addition, ten samples each of blood, vaginal fluid and menstrual blood were obtained; blood and vaginal fluid stains were made in the same manner as used for the semen stains; menstrual blood stain was made by clipping 1 cm^2^ sanitary towel. Samples were obtained from the Center of Medicine Expertise of Sun Yat-Sen University. Finally, the RNA of the all samples including 45 semen, 10 blood, vaginal fluid and menstrual blood was extracted and quantified by UV-2450 (Shimadzu, Japan). All volunteers were anonymous and specimens were obtained under informed consent. The primer and probe sequences of the target genes *miR10b*, *miR135b* and the reference gene *RNU6b* were obtained from the miRBase database (http://www.mirbase.org/index.shtml/); a real-time quantitative PCR kit (TaqMan MicroRNA Assay kit) was purchased from the U.S Applied Biosystems (ABI, Foster city, USA).

The reverse transcription and RT-qPCR reactions were performed according to the TaqMan MicroRNA Assay kit manual, to take 1.5μl RNA template carried out the reverse transcription (see S1 Table). The Assay-ID of *hsa-miR-10b* marker: hsa002218_m1 (primer: RT2218, TaqMan probe: TM2218); The Assay-ID of *hsa-miR-135b* marker: hsa002261_m1 (primer: RT2261, TaqMan probe: TM2261. The expression levels of *miR10b*, *miR135b*, and *RNU6b* in 10 semen samples which be extracted from 40 samples randomly, 10 menstrual blood, 10 peripheral blood, 10 vaginal fluid stains and negative control were detected duplicate 3 times using RT-qPCR, the protocol for the RT-qPCR components in each tube: Premix Ex Taq (2×) 10μl, TaqMan Micro RNA Assay kit Real-time primer-probe (20×), 1.0μl, ROX Reference Dye II (50×) 0.4μl, ddH_2_O 6.6μl, Reverse transcription product 2.0μl, the parameters of cycles 50°C 2min, 95°C 10min, then 95°C 15s, 60°C 60s, 50 cycles at ABI 7500 thermal cycler, and the differences in expression levels were compared. The sensitivity was tested with a serial dilution RNA of semen stains sample at the same time, the concentration from 100ng to 0.001ng/ml. The reliability of method was tested with known 30 normal semen stains, 5 no sperm semen samples and 10 old each kind of semen samples, 1 day, 1 month, 3 months, 6 months and 1 years respectively. Then, those samples were analyzed respectively with RT-qPCR and compared C_t_ values.

The test results were analyzed using the SPSS version 17.0 statistical software package. (Chicago, IL, USA). The means and the standard deviations of the expression of *miR10b*, *miR135b* and *RNU6b* in different body fluids were calculated. The differences were compared, and judgment criteria were established, the level of significance was p< 0.05 for all statistical analyses.

Ethics Statement: The research protocol was approved by the Human Subjects Committee at the Zhongshan School of Medicine, Sun Yat-sen University. Written informed consent was attained by all participants or guardians involved in the study. The research protocol was also approved by the Animal Subjects Committee at the Zhongshan School of Medicine, Sun Yat-sen University.

## Results

### 1. The Expression of *miR10b*, *miR135b*, and *RNU6b* in Body Fluid Stains

The fluorescence detection threshold of *miR10b*, *miR135b*, and *RNU6b* was set at 0.04; at this point, the amplification curves of *miR10b*, *miR135b*, and *RNU6b* are in the exponential growth phase. The expression levels of *miR10b*, *miR135b*, and *RNU6b* were detected to obtain a C_T_ value of body fluid stains. The ΔC_T_ values reflect the C_T_ value of the target gene minus the C_T_ value of the reference gene. The results are shown in Table 1.

**Table 1:** The C_T_ and ΔCт values of miRNA markers that tested by RT-PCR in different body fluid stains.

Type	Sample	Cт			ΔCт	
samples	name	miR10b	miR135b	RNU6b	[10b-U6]	[135b-U6]
Normal sperm semen stains*	S01	28.827	29.705	36.982	-8.155	-7.277
	S03	29.14	29.045	37.419	-8.279	-8.374
	S04	26.566	27.748	35.775	-9.209	-8.027
	S07	26.649	25.618	35.473	-8.825	-9.856
	S09	25.954	25.285	33.68	-7.726	-8.395
	S10	32.516	32.489	38.772	-6.256	-6.283
	S13	26.688	26.576	35.75	-9.062	-9.174
	S21	25.848	25.261	33.475	-7.627	-8.214
	S22	25.287	23.917	33.045	-7.758	-9.128
	S29	26.476	25.791	34.876	-8.4	-9.085
No sperm semen stains	AS01	29.055	27.51	36.106	-7.051	-8.596
	AS02	26.584	26.484	33.291	-6.707	-6.807
	AS03	28.772	30.083	38.248	-9.476	-8.165
	AS04	29.816	29.867	38.112	-8.296	-8.245
	AS05	27.357	27.056	35.498	-8.141	-8.442
	V01	28.068	32.027	32.872	-4.804	-0.845
	V02	33.313	30.698	38.898	-5.585	-8.2
	V03	29.985	30.258	29.767	0.218	0.491
Vaginal swabs stains	V04	29.003	27.803	31.359	-2.356	-3.556
	V05	29.216	27.608	30.518	-1.302	-2.91
	V06	30.491	29.436	32.303	-1.812	-2.867
	V07	32.664	33.74	31.575	1.089	2.165
	V08	34.622	30.747	36.553	-1.931	-5.806
	V09	33.395	32.983	34.925	-1.53	-1.942
	V10	29.411	28.32	32.122	-2.711	-3.802
	PB01	36.916	35.959	34.708	2.207	1.251
	PB02	39.968	37.302	38.709	1.259	-1.41
	PB03	33.534	33.29	32.125	1.409	1.165
Peripheral marks stains	PB04	33.238	32.984	31.225	2.014	1.759
	PB05	35.688	33.91	33.411	2.277	0.499
	PB06	33.314	32.905	32.574	0.74	0.331
	PB07	33.939	32.237	32.349	1.59	-0.112
	PB08	34.88	34.609	33.287	1.593	1.322
	PB09	32.542	33.395	32.3	0.242	1.094
	PB10	32.143	31.823	31.175	0.968	0.648
	MB01	27.306	29.261	33.737	-6.431	-8.914
	MB02	26.976	28.567	35.349	-8.373	-4.476
	MB03	27.637	29.395	32.292	-4.655	-6.781
Menstrual bloodstains stains	MB04	28.628	30.555	33.425	-4.797	-2.896
	MB05	27.517	28.033	34.158	-6.641	-2.871
	MB06	29.707	30.755	34.712	-5.005	-6.125
	MB07	25.812	26.475	29.489	-3.678	-3.957
	MB08	26.452	27.65	32.646	-6.193	-3.015
	MB09	26.267	27.375	31.625	-5.358	-4.995
	MB10	26.243	25.95	35.157	-6.431	-4.25
NTC**	N	undetermined	40.642	undetermined	——	——

Note *: The tested 10 semen samples were extracted from 40 normal semen samples randomly.

** NTC: negative control.

### *2*. The expression levels of *miR10b*, *miR135b*, and *RNU6b*, the specificity and sensitivity of the method

#### 2.1 The expression level of *miR10b*, *miR135b*, and *RNU6b* in different body fluids

**Fig 1:**
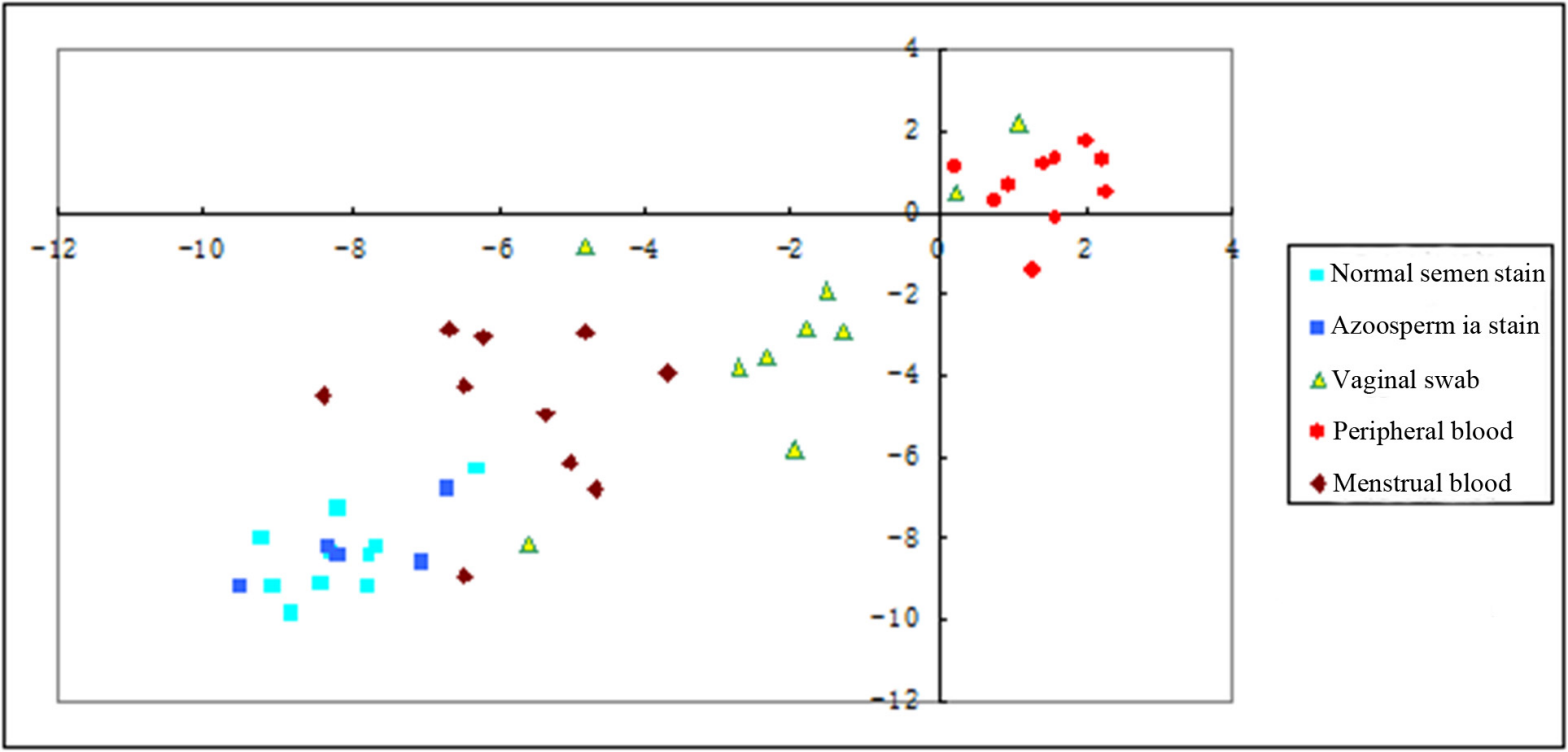
The two-dimensional scatter plots was created with Δ*C*_t_ values. The Δ*C*_t_ values were used to create two-dimensional (2D) scatter plots to determine whether differentiation of each body fluid would be possible. Significantly, distinct clustering of each body fluid was observed in each assay clearly separated from the other body fluid expression data. Individual body fluid data points are represented by colored different shape: brilliant green, normal semen stains; blue, azoospermia stains; green triangle, vaginal swab; red, peripheral blood; brown, menstrual blood.

The value of ΔC_T_ [*10b-U6*] was used as the abscissa axis, and the value of ΔC_T_ [*135b-U6*] was used as the longitudinal axis; the point distribution graph was obtained according to the detection results for different body fluid stains (Fig 1). The figure have show that the scatter-point distributions of the peripheral blood, semen stains, vaginal swabs, and menstrual bloodstains have obvious different. The scattered points for semen stains, vaginal swabs, and menstrual bloodstains were in the same quadrant, but there were characteristic cluster in different fluid stains. The azoosperma (semen stains without sperm) and normal semen stains were found to be distributed in the same area, with no significant differences in location,(mean ΔC_T-_8.1297V-7.9342, p>0.05; -8.3813V-8.051, p>0.05) (see S2 Table).

#### 2.2 The specificity of the method

**Fig 2:**
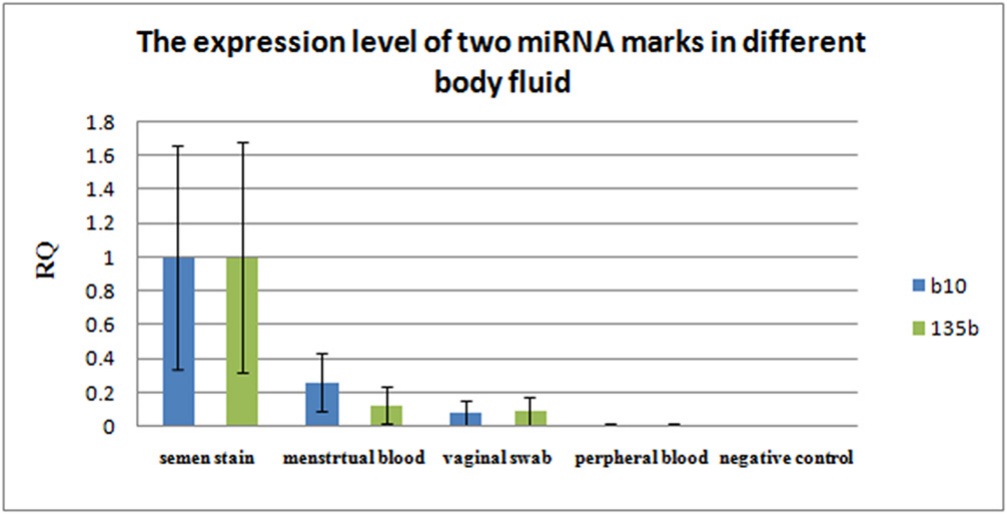
The expression of two miRNA marks in different body fluid. The level of expression are on the X axis, and the relative quantitation (RQ) is on the Y axis. RQ value of *10b* and *135b* is the highest in semen stain, almost 100%, next, it is in menstrual blood and vaginal swab stains. RQ value is the lowest in peripheral blood except negative control.

The histogram was created by comparing the relative expression levels (Relative Quantitation, RQ) of *miR10b* and *miR135b* in different body fluids stains (Fig 2), which showed that the expression levels of *miR10b* and *miR135b* in semen stains were higher than that in the other body fluid stains. To compared C_t_ between semen stain and others body fluid (vaginal swabs, Va; peripheral blood, Pe; menstrual bloodstains, Me) with statistical method to two marke, mean △C_t_ at m10b-8.03195v-2.0724(Va), p<0.001; 1.4299(Pe), p<0.001–5.7562(Me), p<0.002; at 135b-8.2172v-2.7272(Va), p<0.001; 0.6547(Pe), p<0.001; -4.8282(Me), p<0.003 (see S3 Table).

#### 2.3 The sensitivity of the method

The results for the sensitivity of the method are shown in Table 2, which were obtained after the semen samples were a series diluted.

**Table 2:** The results of miRNA RT-qPCR with a series of diluted RNA templates.

Total RNA	Cт			ΔCт		+/-
(ng)	miR10b	miR135b	RNU6b	[10b-U6]	[135b-U6]	
100	27.392	26.29	35.942	-8.55	-9.652	+
10	30.327	29.68	38.968	-8.641	-9.288	+
1	31.788	33.449	undetermined	——	——	Failed*
0.1	32.199	36.752	undetermined	——	——	Failed
0.01	31.938	49.227	undetermined	——	——	Failed
0.001	32.381	undetermined	undetermined	——	——	Failed
NTC**	undetermined	undetermined	undetermined	——	——	Failed

Note *: Failed: When test results of reference gene is undetermined, it is considered that RNA extraction is unsuccessful or the concentration of RNA is too low to be amplified.

** NTC: negative control.

### 3. The Reliability and Detection Capability of the Method

Semen stains stored at different times were detected. The results are shown in Table 3. At 25°C, there are no significant differences in the test results.

**Table 3:** The summarized results of miRNA RT-qPCR with old semen stains.

Storage time at room temperature	Number of samples	positive
1 day	10	10
1 month	10	7
3 months	10	9
6 months	10	8
1 year	10	8

Thirty samples of normal semen stains and five samples of no sperm semen stains were detected. The tested results of those samples were confirmed with the standards defined by the research. The results are 31 confirmed and 3 exclude in 35 semen stains samples, 10 blood, 9 vaginal swabs and 9 menstrual bloodstains exclude. The results were normal, with the exception that the tested gene was not detected in one sample; the results, therefore, suggest that the method is reliable. The old semen samples which were kept in reserve within 1 year could be tested basically (see S4 Table).

## Discussion

MiRNA fragments are short and degrade relatively slowly [5]. Their distributions have the tissue specificity; as such, their unique molecular characteristics may be used as a new way of identifying body fluid stains in forensics. This has important implications regarding the detection of problematic samples (i.e. degraded or old samples) and therefore has been a subject of interest by forensic researchers [6–14].

The results demonstrate that the expression levels of the target genes *miR10b* and *miR135b* were much higher than that of the reference gene *RNU6b* in semen stains. The highest levels of expression of *miR10b* and *miR135b* were in semen stains, as the levels were higher than those found in menstrual bloodstains and in vaginal swabs. It had been reported that level of expression of *miR10b* and *miR135b* are higher in semen stains than in saliva stains [7]. By analyzing the results of this study and comparing the differences in the expression of the target genes using statistical methods, criteria can be derived to distinguish a semen stain from other body fluid stains. The standards were defined as follows: when using the RT-qPCR technique to detect an miRNA marker, the threshold value should be set to 0.04; the derived C_T_ value can be detected in the target genes *miR10b* and *miR135b* and in the internal reference gene *RNU6b*, and C_T_ values are<40, when ΔC_T_ [10b-U6]<-5.5, and ΔC_T_ [*135b-U6*]<-6, respectively the semen stains can be confirmed. The confirmation standards was derived from the research results by Statistical method and samples test. A test stain can then be compared to the semen stain. In this study, 35 semen stain samples were analyzed. The results were compared to this standard, with the results falling within the positive range 31 except 3 exclude sperm and 1 RNA degradation or extract unsuccessful. Furthermore, the five semen stains without sperm were analyzed using the same test methods and criteria; the results were also within the positive range. In the same way, ten vaginal swab stain, ten menstrual bloodstains and ten peripheral bloodstains were analyzed, the results have not been found to fall within the positive range except a sample of menstrual bloodstains (MB1) and vaginal swabs stains (V2)(Table 2). The result of the two samples was exceptional, and the reasons were relatively complicated. One of a reason was the error caused by operator during the experiment, another perhaps was abnormal expression of the sample itself, such as, a influence was brought about by some unknown disease. It needs to be further researched. Therefore, the technology can be used to distinguish normal semen stains and without sperm stains from other bodily fluids. These results are concordant those of Hanson’s [7], who suggested that the expression levels of *miR10b* and *miR135b* were lower than that of *RNU6b* in a semen stain, but theirs expression levels were higher than other body fluid stains. Accordingly, the expression levels of *miR10b* and *miR135b* were suitable for the specificity markers of miRNA to confirm semen stains [7, 11]. However, this experimental result demonstrates that the expression levels of *miR10b* and *miR135b* were much higher than that of the reference gene *RNU6b* in semen; these results are inadequacy conformity with Hansons’ conclusions. Why did the results of this study were partly different from Hanson? The real reason have not been figure out, maybe was condition of experiment, or sequence of probe and primer, or taken sample different. It needs to be research further.

Although most of the semen stains and non-semen stains can be distinguished by the use of the miRNA RT-qPCR technique with a strict positive standard, the false-positive and negative rates when using this technique are still higher than that of the mRNA RT-qPCR technique. This is one particular drawback that is worthy of further attention and research. Theoretically, detecting miRNA could have higher sensitivity than mRNA because of its wider distribution and shorter fragments [10, 12, 14]. Actually, our experimental results illustrated also that the detection sensitivity of the miRNA RT-qPCR techniques to detect a semen stain is within the 10ng level, which is lower than that of the mRNA RT-qPCR technique(data do not be shown). But, by analyzing the experimental data, it was found that miRNA cannot be detected in low concentration semen stains; this was mainly due to the fact that the internal reference gene of *RNU6b* was not detected. *miR10b* and *miR135b* have good amplification curves, with a C_T_ value that is negatively correlated with the template concentration. The sensitivity of the miRNA RT-qPCR technique to detect semen stains will increase if an internal control gene with a relatively higher expression level is used.

In this experiment, the old semen stains were identified using the semen stain confirmation miRNA RT-qPCR system after one day, one month, three months, six months, and one year at 25°C (under dark and dry conditions). The results demonstrated that the positive rates of old stain preservation at one year were 80%. The samples were stored for different times, but the expression levels of *miR10b*, *miR135b*, and *RNU6b* did not significantly vary among different research times. Therefore, it was concluded from the studied results that the stability of miRNA markers was better than that of mRNA (About comparing the stability and sensitivity of miRNA markers and mRNA markers, please read detailed data http://202.116.65.75/c/portal/layout?pl_id=PUB.1001.219 for reference, 《A study of specific mRNA and microRNA markers in seminal stains》 author: Tianyu Xue, a master's thesis.) As such, this technique is more suitable for the identification of old or degraded body fluid stain samples. The further experiment is necessary when the samples were laid up for more lengthy time.

In short, the use of quantitative PCR technology makes it possible to use the expression of *miR10b* and *miR135b* as a detection marker in semen stains, especially when used for old or degraded samples.

## Supporting Information

S1 Table The protocol for the reverse transcription components and cycles parameters. (DOC)

S2 Table Comparing different of mean △Ct between normal sperm semen stains and no sperm semen stains. (DOC)

S3 Table Comparing different of mean △Ct between normal sperm semen stains and no sperm semen stains. (DOC)

S4 Table The tested result of miRNA markers in old seminal stains. (DOC)
