# Supplementary material for: Investigation of the Application of miR10b and miR135b in the Identification of Semen Stains
Source: PLoS One. 2015 Sep 10;10(9):e0137067. doi: 10.1371/journal.pone.0137067 (PMC4565637; doi:10.1371/journal.pone.0137067)
Supplement: S1 Table — (DOC) [file pone.0137067.s001.doc]

S1 Table : The protocol for the reverse transcription components and cycles parameters

| Reverse transcription components | stock | µl/tube | Final concentration |
| --- | --- | --- | --- |
| 5× RT Buffer | 5× | 2.0 | 1× |
| dNTP Mixture | 10mM | 0.5 | 0.5mM |
| TaqMan® MicroRNA Assay  kit RT primer | 5× | 0.5 | 0.25× |
| M-MLV | 200U/µl | 0.5 | 10U/µl |
| RNase Inhibitor | 40 U/µl | 0.2 | 0.8U/µl |
| Nuclease-free water | - | 4.8 | - |
| RNA template | - | 1.5 | - |

Final - 10µl -

The parameters of cycles: 16℃ 30min, 42℃ 30min, 85℃ 5min, 4℃ forever at ABI 7500 thermal cycler.
