# Supplementary material for: Investigation of the Application of miR10b and miR135b in the Identification of Semen Stains
Source: PLoS One. 2015 Sep 10;10(9):e0137067. doi: 10.1371/journal.pone.0137067 (PMC4565637; doi:10.1371/journal.pone.0137067)
Supplement: S2 Table — (DOC) [file pone.0137067.s002.doc]

S2 Table : Comparing different of mean △Ct between normal sperm semen stains and no sperm semen stains

| Normal sperm semen stains | |  | No sperm semen stains | |
| --- | --- | --- | --- | --- |
| [10b-U6] | [135b-U6] |  | [10b-U6] | [135b-U6] |
| -8.155 | -7.277 |  | -7.051 | -8.596 |
| -8.279 | -8.374 |  | -6.707 | -6.807 |
| -9.209 | -8.027 |  | -9.476 | -8.165 |
| -8.825 | -9.856 |  | -8.296 | -8.245 |
| -7.726 | -8.395 |  | -8.141 | -8.442 |
| -6.256 | -6.283 |  | -39.671 | -40.255 |
| -9.062 | -9.174 |  | -7.9342* | -8.051* |
| -7.627 | -8.214 |  |  |  |
| -7.758 | -9.128 |  |  |  |
| -8.4 | -9.085 |  |  |  |
| -81.297 | -83.813 |  |  |  |
| -8.1297* | -8.3813* |  |  |  |

* are mean △Ct.
