# Supplementary material for: Investigation of the Application of miR10b and miR135b in the Identification of Semen Stains
Source: PLoS One. 2015 Sep 10;10(9):e0137067. doi: 10.1371/journal.pone.0137067 (PMC4565637; doi:10.1371/journal.pone.0137067)
Supplement: S3 Table — (DOC) [file pone.0137067.s003.doc]

S3 Table :Comparing different of mean △Ct between normal sperm semen stains and no sperm semen stains

| Normal sperm semen and no sperm semen stains | |  | Vaginal swabs stain | |  | Peripheral blood stains | |  | Menstrual blood stains | |
| --- | --- | --- | --- | --- | --- | --- | --- | --- | --- | --- |
| [10b-U6] | [135b-U6] |  | [10b-U6] | [135b-U6] |  | [10b-U6] | [135b-U6] |  | [10b-U6] | [135b-U6] |
| -8.155 | -7.277 |  | -4.804 | -0.845 |  | 2.207 | 1.251 |  | -6.431 | -8.914 |
| -8.279 | -8.374 |  | -5.585 | -8.2 |  | 1.259 | -1.41 |  | -8.373 | -4.476 |
| -9.209 | -8.027 |  | 0.218 | 0.491 |  | 1.409 | 1.165 |  | -4.655 | -6.781 |
| -8.825 | -9.856 |  | -2.356 | -3.556 |  | 2.014 | 1.759 |  | -4.797 | -2.896 |
| -7.726 | -8.395 |  | -1.302 | -2.91 |  | 2.277 | 0.499 |  | -6.641 | -2.871 |
| -6.256 | -6.283 |  | -1.812 | -2.867 |  | 0.74 | 0.331 |  | -5.005 | -6.125 |
| -9.062 | -9.174 |  | 1.089 | 2.165 |  | 1.59 | -0.112 |  | -3.678 | -3.957 |
| -7.627 | -8.214 |  | -1.931 | -5.806 |  | 1.593 | 1.322 |  | -6.193 | -3.015 |
| -7.758 | -9.128 |  | -1.53 | -1.942 |  | 0.242 | 1.094 |  | -5.358 | -4.995 |
| -8.4 | -9.085 |  | -2.711 | -3.802 |  | 0.968 | 0.648 |  | -6.431 | -4.25 |
| -7.051 | -8.596 |  |  |  |  |  |  |  |  |  |
| -6.707 | -6.807 |  |  |  |  |  |  |  |  |  |
| -9.476 | -8.165 |  |  |  |  |  |  |  |  |  |
| -8.296 | -8.245 |  |  |  |  |  |  |  |  |  |
| -8.141 | -8.442 |  |  |  |  |  |  |  |  |  |
| -8.03195 | -8.2712 |  | -2.0724 | -2.7272 |  | 1.4299 | 0.6547 |  | -5.7562 | -4.828 |
|  |  |  | P<0.001 | P<0.001 |  | P<0.001 | P<0.001 |  | P<0.002 | P<0.003 |
