# Supplementary material for: Investigation of the Application of miR10b and miR135b in the Identification of Semen Stains
Source: PLoS One. 2015 Sep 10;10(9):e0137067. doi: 10.1371/journal.pone.0137067 (PMC4565637; doi:10.1371/journal.pone.0137067)
Supplement: S4 Table — (DOC) [file pone.0137067.s004.doc]

S4 Table ：The tested result of miRNA markers in old seminal stains

| Sample name | Cт | | |  | ΔCт | | +/- |
| --- | --- | --- | --- | --- | --- | --- | --- |
|  | miR10b | miR135b | RNU6b |  | [10b-U6] | [135b-U6] |  |
| ST01（1d） | 23.541 | 23.211 | 31.498 |  | -7.957 | -8.287 | + |
| ST02（1d） | 24.897 | 23.898 | 31.376 |  | -6.479 | -7.478 | + |
| ST03（1d） | 29.140 | 29.045 | 37.419 |  | -8.279 | -8.374 | + |
| ST04（1d） | 32.516 | 32.489 | 38.772 |  | -6.256 | -6.283 | + |
| ST05（1d） | 23.343 | 21.763 | 31.391 |  | -8.048 | -9.628 | + |
| ST06（1d） | 27.034 | 23.810 | 32.574 |  | -5.540 | -8.764 | + |
| ST07（1d） | 28.184 | 25.564 | 37.465 |  | -9.281 | -11.901 | + |
| ST08（1d） | 26.688 | 26.576 | 35.750 |  | -9.062 | -9.174 | + |
| ST09（1d） | 24.325 | 22.408 | 31.790 |  | -7.465 | -9.382 | + |
| ST10（1d） | 26.476 | 25.791 | 34.876 |  | -8.400 | -9.085 | + |
| ST01（1m） | 27.045 | 25.806 | 32.572 |  | -5.527 | -6.766 | + |
| ST02（1m） | 26.583 | 25.412 | 32.460 |  | -5.877 | -7.048 | + |
| ST03（1m） | 37.045 | 34.477 | 36.774 |  | 0.271 | -2.297 | - |
| ST04（1m） | 25.464 | 23.876 | 31.173 |  | -5.709 | -7.297 | + |
| ST05（1m） | 27.017 | 22.764 | 32.780 |  | -5.763 | -10.016 | + |
| ST06（1m） | 32.542 | 28.508 | 34.600 |  | -2.058 | -6.092 | - |
| ST07（1m） | 29.903 | 30.088 | 33.470 |  | -3.567 | -3.382 | - |
| ST08（1m） | 25.238 | 21.882 | 33.454 |  | -8.216 | -11.572 | + |
| ST09（1m） | 29.334 | 27.389 | 36.093 |  | -6.759 | -8.704 | + |
| ST10（1m） | 24.579 | 22.085 | 33.324 |  | -8.745 | -11.239 | + |
| ST01（3m） | 25.501 | 23.268 | 33.364 |  | -7.863 | -10.096 | + |
| ST02（3m） | 25.337 | 23.649 | 31.889 |  | -6.552 | -8.240 | + |
| ST03（3m） | 24.835 | 21.767 | 33.695 |  | -8.860 | -11.928 | + |
| ST04（3m） | 25.180 | 24.813 | 31.500 |  | -6.320 | -6.687 | + |
| ST05（3m） | 25.044 | 25.574 | 32.150 |  | -7.106 | -6.576 | + |
| ST06（3m） | 31.685 | 28.793 | 34.443 |  | -2.758 | -5.650 | - |
| ST07（3m） | 25.549 | 24.755 | 31.501 |  | -5.952 | -6.746 | + |
| ST08（3m） | 25.283 | 22.718 | 34.257 |  | -8.974 | -11.539 | + |
| ST09（3m） | 24.600 | 23.448 | 32.163 |  | -7.563 | -8.715 | + |
| ST10（3m） | 25.225 | 22.700 | 32.004 |  | -6.779 | -9.304 | + |
| ST01（6m） | 25.501 | 24.182 | 33.121 |  | -7.620 | -8.939 | + |
| ST02（6m） | 24.587 | 23.595 | 31.327 |  | -6.740 | -7.732 | + |
| ST03（6m） | 24.519 | 23.101 | 33.334 |  | -8.815 | -10.233 | + |
| ST04（6m） | 25.192 | 23.602 | 33.231 |  | -8.039 | -9.629 | + |
| ST05（6m） | 25.698 | 24.006 | 32.779 |  | -7.081 | -8.773 | + |
| ST06（6m） | 34.662 | 31.685 | 35.904 |  | -1.242 | -4.219 | - |
| ST07（6m） | 26.892 | 26.550 | 32.668 |  | -5.776 | -6.118 | + |
| ST08（6m） | 25.327 | 23.194 | 34.098 |  | -8.771 | -10.904 | + |
| ST09（6m） | 25.444 | 23.825 | 32.548 |  | -7.104 | -8.723 | + |
| ST10（6m） | 29.586 | 26.063 | 33.369 |  | -3.783 | -7.306 | - |
| ST01（1y） | 24.979 | 23.573 | 33.810 |  | -8.831 | -10.237 | + |
| ST02（1y） | 25.295 | 25.588 | 32.461 |  | -7.166 | -6.873 | + |
| ST03（1y） | 31.763 | 28.759 | 39.198 |  | -7.435 | -10.439 | + |
| ST04（1y） | 27.497 | 28.376 | 34.825 |  | -7.328 | -6.449 | + |
| ST05（1y） | 25.731 | 24.097 | 34.886 |  | -9.155 | -10.789 | + |
| ST06（1y） | 36.591 | 35.816 | 39.368 |  | -2.777 | -3.552 | - |
| ST07（1y） | 25.775 | 29.983 | 34.815 |  | -9.040 | -4.832 | - |
| ST08（1y） | 25.541 | 23.787 | 33.975 |  | -8.434 | -10.188 | + |
| ST09（1y） | 28.956 | 27.932 | 37.592 |  | -8.636 | -9.660 | + |
| ST10（1y） | 28.530 | 26.376 | 35.375 |  | -6.845 | -8.999 | + |
